# Improved therapeutic effectiveness by combining liposomal honokiol with cisplatin in lung cancer model

**DOI:** 10.1186/1471-2407-8-242

**Published:** 2008-08-16

**Authors:** Qi-qi Jiang, Lin-yu Fan, Guang-li Yang, Wen-Hao Guo, Wen-li Hou, Li-juan Chen, Yu-quan Wei

**Affiliations:** 1 State Key Laboratory of Biotherapy and Cancer Center, West China Hospital, West China Medical School, Sichuan University, Chengdu, Sichuan, PR China; 2 Dept of Respiratory Diseases and Xuan Wu Lung Cancer Center, Xuan Wu hospital of Capital Medical University, Beijing, PR China

## Abstract

**Background:**

Honokiol is a major bioactive compound extracted from Magnolia. The present study was designed to determine whether liposomal honokiol has the antitumor activity against human lung cancer as well as potentiates the antitumor activity of cisplatin in A549 lung cancer xenograft model, if so, to examine the possible mechanism in the phenomenon.

**Methods:**

human A549 lung cancer-bearing nude mice were treated with liposomal honokiol, liposomal honokiol plus DDP or with control groups. Apoptotic cells and vessels were evaluated by fluorescent in situ TUNEL assay and by immunohistochemistry with an antibody reactive to CD31 respectively.

**Results:**

The present study showed that liposomal honokiol alone resulted in effective suppression of the tumor growth, and that the combined treatment with honokiol plus DDP had the enhanced inhibition of the tumor growth and resulted in a significant increase in life span. The more apparent apoptotic cells in the tumors treated with honokiol plus DDP was found in fluorescent in situ TUNEL assay, compared with the treatment with control groups. In addition, the combination of honokiol and DDP apparently reduced the number of vessels by immunolabeling of CD31 in the tissue sections, compared with control groups.

**Conclusion:**

In summary, our data suggest that honokiol alone had the antitumor activity against human lung cancer in A549 lung cancer xenograft model, and that the combination of honokiol with DDP can enhance the antitumor activity, and that the enhanced antitumor efficacy in vivo may in part result from the increased induction of the apoptosis and the enhanced inhibition of angiogenesis in the combined treatment. The present findings may be of importance to the further exploration of the potential application of the honokiol alone or the combined approach in the treatment of lung carcinoma.

## Background

Lung cancer is the leading cause of cancer related death in both males and females. Non-small cell lung cancer (NSCLC) comprises approximately 75–80% of all lung cancers. Despite aggressive approaches made in the therapy of lung cancer in the past decades, the prognosis of NSCLC remains poor, with 5-year survival rates of 5–14%, even if treated with surgery, radiotherapy and/or chemotherapy [1-3]. Efforts are therefore continuing to develop new and less toxic therapeutic approaches for the treatment of lung cancer.

Honokiol is a major bioactive compound extracted from Magnolia [4]. It has been reported that honokiol induces apoptosis and inhibits the in vitro growth of a variety of human cancer cell lines such as leukemia cell lines HL-60 and Molt 4B; colon cancer cell line RKO; lung cancer cell line CH27 [5-9]. Honokiol also exhibited the potent anti-proliferative activity against endothelial cells transformed endothelial cell line SVR and the primary human endothelial cells in vitro [4]. These findings suggest that honokiol has both anti-angiogenic and anti-tumor activity in vitro. Furthermore, there are several reports of in vivo antitumor activity of honokiol against skin tumors and SVR angiosarcoma in a mouse model [4,10]. However, little is known about the antitumor activity in lung cancer in an animal model.

Cisplatin (DDP) remains the most widely used first-line element of cytotoxic chemotherapy to solid tumors [11]. However, the efficacy of DDP based on the treatment is limited in curing most solid tumors due to dose-dependent toxicity. Therefore, several new therapeutic strategies under investigation involve modulation of cellular chemosensitivity, including the inhibition of tumor neovascularization, reversing tumor resistance, and increasing therapeutic effects of chemotherapy [12,13].

Because of differences in the possible mechanisms of action and toxicity profiles of honokiol and cisplatin, the combination of the above two agents may have clinical potential. The present study was designed to determine whether liposomal honokiol has the antitumor activity against human lung cancer as well as potentiates the antitumor activity of cisplatin in A549 lung cancer xenograft model, if so, to examine the possible mechanism in the phenomenon, and to provide some potential implications for the treatment of human lung cancer.

## Methods

### Agents

Polyethylene glycol 4000-phosphoethanolamine (PEG-PE), cholesterol and PC were purchased from Sigma Chemical Co, (St. Louis, MO). Cisplatin (DDP) was purchased from QiLu pharmaceutical factory. A rat anti-mouse CD31 monoclonal antibody was purchased from BD Biosciences Co (PharMingen, San diego, CA). In situ Cell Death Detection kit was purchased from Roche Co. (Promega, Madison, WI). Honokiol was separated and purified by High-speed counter-current chromatography from cortex Magnoliae Officinalis, as reported previously [14]. Its purity and identification were analyzed by high performance liquid chromatography and nuclear magnetic Resonance. The purities of the isolated honokiol were more than 99.6%.

### Preparation of liposomal honokiol

The preparation of PEG-modified Liposomal honokiol was modified as reported previously by us [15]. Briefly, the mixture of PC, cholesterol, PEG4000-PE and honokiol in weight ratios of 1:0.15:0.24:0.22 were dissolved in 15 ml chloroform/methanol at a ratio of 3:1 (v/v). The mixture was gently warmed to 40°C in a round-bottomed flask, and the solvent was evaporated under vacuum in a rotary evaporator until a thin lipid film was formed. The dried lipid films were left overnight and sonicated in 5% glucose solution at constant container followed by concentrated and lyophilized. The preparation of empty free liposome was the same way as the liposomal honokiol withour honokiol addition. The final liposomal honokiol was small multilamellar liposomes in a size range of 130 ± 20 nm. Lyophilized liposomal honokiol and free liposome were dissolved in 5% glucose water for vitro and vivo studies.

### Animal tumor models and treatment

Human A549 lung adenocarcinoma cells were obtained from the American Type Culture Collection (ATCC, Manassas, VA). The cells were maintained in RPMI1640 (Life Technologies, Bedford, MA) containing 10% heat-inactivated FCS, 100 units/mL penicillin, and 100 units/mL streptomycin in a humid 5% CO2 incubator at 37°C. Human A549 tumor models were established in 6 – 8 weeks old, athymic nude mice (SPF grade). Athymic nude mice were inoculated with A549 cells/0.1 ml (1.0 × 10^7^) s.c. All these mice were obtained from Sichuan University Animal Center (Sichuan, Chengdu, China). All studies involving mice were approved by the institute's animal care and use committee.

In our preliminary experiment, we have performed a series of experiment in order to determine the optimum doses for the two agents. Human A549 lung cancer cells (1.0 × 10^7^) were injected s.c.. Once tumors were palpable, mice were randomly assigned into each group (n = 5 mice/group). Treatments were given i.p. with PBS, liposome, free honokiol alone, or liposomal honokiol at different doses of 1 to 50 mg/kg every day for 21 days, respectively.

In the next set of the experiment, 549 lung cancer cells (1.0 × 10^7^) were injected s.c.. Seven days later, once tumors were palpable, mice were randomly divided into each group. Treatments were given i.p. with liposomal honokiol (25 mg/kg) every day for 21 days or with PBS, liposome or cisplatin (5 mg/kg) or free honokiol alone as a control. In combination treatment, liposomal honokiol is injected 4 hours later after cisplatin administration. DDP (5 mg/kg) was given i.p. twice a week for two weeks, as we reported previously [13]. In the other experiments, the courses are repeated every 4 weeks, namely, treatment was stopped for 1 week followed by a new cycle of therapy. Survival time and tumor volume were observed. Tumor size was determined by caliper measurement of the largest and perpendicular diameters. Tumor volume was calculated according to the formula V = 0.52*ab*^2 ^[14].

### Pharmacokinetic studies

Pharmacokinetic studies were performed as reported previously by us [15]. Briefly, on day 15 after the inoculation of the tumor, the liposomal honokiol or the free honokiol dissolved in DMSO was given i.v. to these mice at a dose of 25 mg/kg. Mice were sacrificed at defined time points (at 5 and 30 min, and at 1, 5, 12, 24, and 48 h after the administration of honokiol). At each time point, four mice were sacrificed and their blood was collected from the orbital cavity vein plexus, heparinized, and centrifuged to obtain the plasma. The tumor, kidney, liver, lung, intestine, heart, and spleen tissues were excised and weighed, and homogenized. Plasma and the homogenized organs were added to accetonitrile, and supernatant fluid was collected and evaporated to dryness. The dry residues were dissolved in methanol for high-performance liquid chromatography analyses.

### Quantitative assessment of apoptosis

Quantitative assessment of apoptosis was performed as reported previously by us [13,15]. Briefly, terminal deoxynucleotidyl transferase – mediated nick-end labeling staining was done using an in situ cell death detection kit (Roche Molecular Biochemicals) following the manufacturer's protocol. It is based on the enzymatic addition of digoxigenin nucleotide to the nicked DNA by terminal deoxynucleotidyl transferase. In tissue sections, five equal-sized fields were randomly chosen and analyzed. Density was evaluated in each field, yielding the density of apoptotic cells (apoptosis index).

### Detection of CD31 by immunohistochemistry

The anti-angiogenesis effects were determined by immunohistochemistry with an antibody reactive to CD31 as described previously by us [15]. Briefly, frozen sections were fixed in acetone and incubated with a monoclonal goat anti-rabbit CD31 antibody (1:400; Santa Cruz Biotechnology) at 4°C overnight, followed by incubation with biotinylated polyclonal rabbit anti-rat antibody (1:200; Vector, Burlingame, CA) in a humidified chamber for 1 h, and then were immersed in 0.3% H_2_O_2 _in absolute methanol for 15 min to block endogenous peroxidase. Positive reaction was visualized using 3, 3-diaminobenzidine as chromagen (DAB substrate kit; Vector). Sections were counterstained with hematoxylin and mounted with glass coverslips. Vessel density was determined by counting the number of microvessels per high-power field in the section. Then tissue sections were visualized in an Olympus microscope to determine microvessel density (MVD).

### Observation of potential toxicity

The toxicities of the treatment regimens were estimated by changes in the incidence of drug-associated death. Gross measures, such as weight loss, ruffling of fur, life span, behavior, and feeding were studied. Tissues of heart, liver, spleen, lung, kidney, brain, bone marrow, etc. were also fixed in 10% neutral buffered formalin solution and embedded in paraffin. Sections of 4 μm were stained with H&E. Blood was obtained from the tail vein for complete blood count and differentials as well as enzyme analysis.

### Statistical analysis

For comparison of individual time points, different results between the groups were reported as means ± SD and tested by performing one-way analysis of variance (ANOVA) and a log-rank test. The statistical significance level was set as p less than 0.05.

## Results

### Tumor growth inhibition

**Figure 1:**
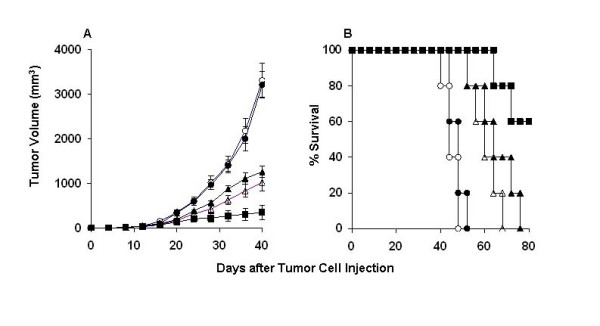
The representative experiment of tumor suppression and survival advantage in mice. Mice were treated i.p. with liposomal honokiol (25 mg/kg) every day for 21 days. In the combination treatment, liposomal honokiol is injected 4 hours later after cisplatin administration. (5 mg/kg, i.p. twice a week for two weeks). In addition, the control groups including PBS, liposome or cisplatin (5 mg/kg) alone. *A*, suppression of s.c. tumor growth in mice. The combination of liposomal honokiol with DDP can enhance the inhibition of the tumor growth. The results were expressed as the mean volume ± SD; *B*, a significant increase in survival in combined treatment mice compared with the controls. Liposomal honokiol plus cisplatin (solid square), liposomal honokiol (open triangle), cisplatin (solid triangle), liposome (open circles) and PBS (solid circle).

In order to determine the optimum doses for honokiol, human A549-bearing nude mice were treated with liposomal honokiol at different doses ranged from 1 to 50 mg/kg. The mice treated with 15 and 10 mg/kg liposomal honokiol showed different inhibiting response to tumor compared with control group, including free liposome group and PBS controls. When the dose of liposomal honokiol was elevated to 25 mg/kg, there are significantly inhibitions of tumor growth, whereas there was no further enhancement of the antitumor activity at the dose of 50 mg/kg. Hence, we selected the dose of 25 mg/kg as optimum dose for the combination treatment with cisplatin. In the next set of experiment, Mice were treated with intraperitoneal injection of liposomal honokiol at 25 mg/Kg once daily for 21 days and/or administration of DDP i.p. twice a week for two weeks (5 mg/kg, starting at 4 hours before the initiation of liposomal honokiol) or appropriate controls 0.2 ml PBS or liposome alone at the same time point. Tumor volume and life span of mice assay showed that both liposomal honokiol and DDP individually resulted in the antitumor activity (Figure 1A and 1B). Combined treatment with liposomal honokiol plus DDP had a superior suppression of the tumor growth antitumor (Figure 1A). For example, the combined group showed markedly regression of tumor volume (352.43 ± 159.98 mm^3^) on the day 40, compared with the control groups, including PBS (3317.28 ± 380.46 mm^3^), liposome (3215.32 ± 295.80 mm^3^), liposomal honokiol (1028.72 ± 190.59 mm^3^) or cisplatin (1264.27 ± 121.12 mm^3^) alone. The combination of liposomal honokiol therapy with DDP also resulted in a significant increase in life span (Figure 1B). Similar results were also found in LLC Lewis lung cancer model in C57BL/6 mice (data not shown).

### Inhibition of angiogenesis by combination Liposomal honokiol plus DDP

**Figure 2:**
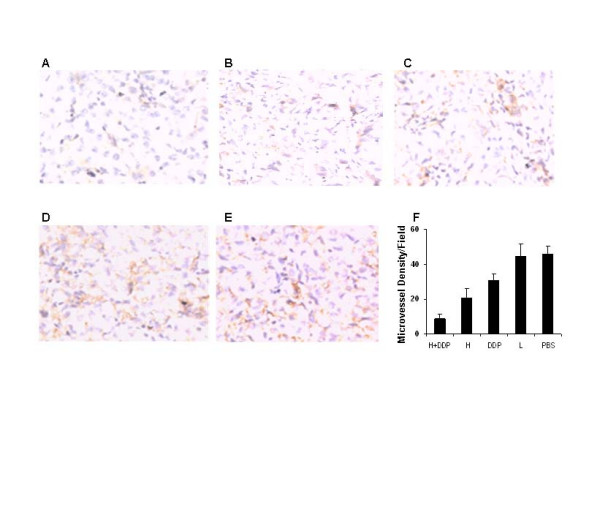
The representative experiment of inhibition of the angiogenesis within tumors. Mice bearing the established tumors were administered i.p. with liposomal honokiol (25 mg/kg) every day for 21 days, and/or cisplatin (5 mg/kg, i.p. twice a week for two weeks).or liposome or PBS alone. Vessel density was determined by counting per high-power field in the sections stained with an antibody reactive to CD31, as described in *Materials and Methods*. The combination of liposomal honokiol and DDP (A) apparently reduced the number of the microvessels, compared with the control groups, including liposomal honokiol (B), cisplatin (C), liposome (D) or PBS (E) alone. Vessel density was determined by counting per high-power (F).*Column*, the mean of the microvessels per high-power field; *bars*, ± SD. H and L mean liposomal honokiol and liposome alone respectively.

Having witnessed apparent antitumor activity in A549 human lung cancer model, we further quantified vessel density as measures of angiogenesis by immunolabeling of CD31 in tissue sections. The combination of liposomal honokiol and DDP apparently reduced the number of vessels compared with control groups (Figure 2), including liposome, PBS, liposomal honokiol, DDP alone (P < 0.05). Angiogenesis was also inhibited in the treatment with honokiol or DDP alone (Figure 2).

### In vivo Induction of apoptosis with the combined Treatment

**Figure 3:**
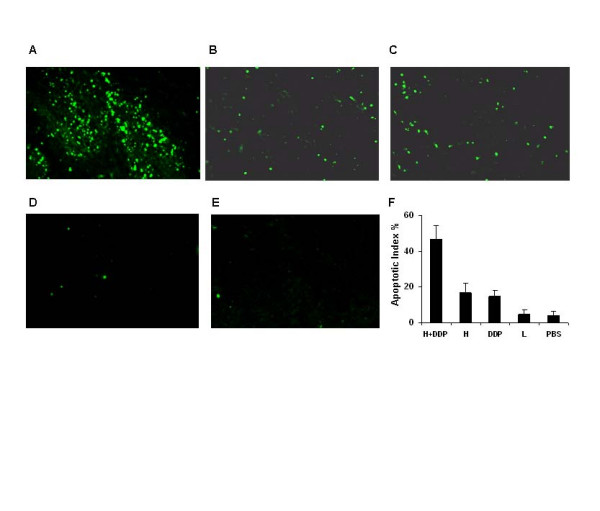
Induction of the apoptosis in vivo. Sections from the tumor-bearing mice treated with liposomal honokiol and DDP (A), liposomal honokiol (B), cisplatin (C), liposome (D) or PBS (E) alone were stained with FITC-dUTP as described in *Materials and Methods*. Apoptotic nuclei (green) were identified by TUNEL staining with FITC-dUTP and observed under a fluorescence microscope (× 200). The treatment with liposomal honokiol and DDP showed apparent apoptotic cells within the tumors, compared treatment with the control groups. Data for the quantitative assessment of apoptosis was expressed as the mean apoptotic index ± SDs (F). H and L mean liposomal honokiol and liposome alone respectively.

To explore the role of liposomal honokiol on the apoptosis of tumor cells, we examined the apoptosis-related molecular marker on tumor sections. An apoptosis detection kit was used to detect early DNA fragmentation associated with apoptosis. The treatment with liposomal honokiol or DDP alone affected the apoptosis rate of tumor cells, whereas the density of the apoptotic cancer cells apparently increased after the combined therapy (Figure 3). Data represent the mean apoptotic index ± SD of cancer cells as percent normalized to apoptotic index of cancer cells.

### The plasma honokiol concentrations of liposomal honokiol and free honokiol

**Figure 4:**
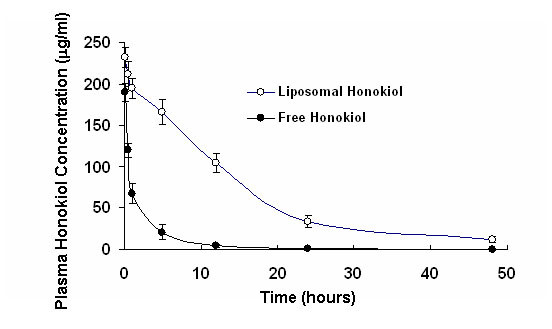
The concentration-time curve of honokiol in plasma both liposomal honokiol and free honokiol. Mice were i.v. treated by liposomal or free honokiol at a dose of 25 mg/kg, as described in Materials and Methods. The honokiol in plasma was detected as described in Materials and Methods. Liposomal honokiol (open circle), free honokiol (solid circle).

Plasma honokiol concentrations in tumor-bearing mice were determined after i.v. injection of 25 mg/kg liposomal honokiol and free honokiol. The pharmacokinetics of liposomal honokiol differed significantly from those of free honokiol. Liposomal honokiol prolonged blood circulation times in tumor-bearing mice in comparison with free honokiol (Figure 4). The plasma honokiol concentrations remained above 30 and 10 μg/ml for 24 and 48 hours study respectively in liposomal honokiol-treated mices, whereas those fell quickly, with less than 5 μg/ml by 12 hours in free honokiol-treated mice,.

### Observation of the potential toxicity

The animals were particularly investigated for potential toxicity. None of pathologic changes in liver, lung, kidney, etc. were found by microscopic examination after the administration of liposomal honokiol or liposomal honokiol plus DDP. No adverse consequences were shown in gross measures, such as weight loss, ruffling of fur, life span, behavior, or feeding. For the evaluation of the effects on hematopoiesis, animals were subjected to complete peripheral blood counts and differentials. Analysis of peripheral blood revealed that no significant difference was observed between DDP-treated and the combined treatment group (Table 1). In addition, the treatment with liposomal honokiol alone did not show the decrease of blood cells, compared with saline control group. Our data suggested that the traditional chemotherapeutic DDP in the combination with honokiol may be concurrently practicable with less host toxicity.

**Table 1:** The representative experiment of hematologic evaluation

	RBC (10^6^/μl)	Platelets(10^6^/μl)	WBC(10^3^/μl)	Neutrophils(10^3^/μl)	Lymphocytes(10^3^/μl)
Saline	10.8 ± 1.7	1.6 ± 0.2	10.8 ± 3.2	4.1 ± 2.2	4.6 ± 2.5
Honokiol	9.2 ± 2.1	1.6 ± 0.4	10.2 ± 2.4	4.0 ± 1.5	4.2 ± 1.8
DDP	9.2 ± 2.6	1.5 ± 0.5	8.9 ± 2.1	3.6 ± 1.3	3.2 ± 2.3
Honokiol					
plus DDP	9.1 ± 3.3	1.5 ± 0.7	8.8 ± 2.8	3.8 ± 1.7	3.3 ± 1.9

RBC, platelets, WBC, neutrophils and lymphocyte counts were performed before therapy and at weekly intervals for 4 weeks. The administration of liposomal honokiol plus DDP was described in the Materials and Methods section, The treatment with honokiol, DDP or the combined did not show the significant decrease of the cell counts on day 14 after the therapy, compared with saline alone(P > 0.05), as shown in the table. The similar results were found on day 7, 21, 28 after the therapy.

## Discussion

It has been reported that honokiol induces apoptosis and inhibits the in vitro growth of a variety of human cancer cell lines [5-9]. Honokiol also shows the potent anti-proliferative activity against endothelial cells transformed endothelial cell line SV and the primary human endothelial cells in vitro [4]. These findings suggest that honokiol has both anti-angiogenic and anti-tumor activity. In the present study, several observations have been made concerning the induction of apoptosis, the inhibition of angiogenesis, antitumor activity and the combined treatment with liposomal honokiol plus DDP. The present study has, to our knowledge, for the first time demonstrated that liposomal honokiol had the inhibitory effect on the *in vivo *growth of human lung cancer in A549 lung cancer xenograft model, and that liposomal honokiol plus DDP induced the enhanced antitumor activity and augmented the induction of apoptosis in lung cancer cells in vivo as well as the inhibition of the angiogenesis.

Although the exact mechanism by which the combination of liposomal honokiol with DDP can enhance the antitumor activity remained to be determined, the enhanced antitumor efficacy in vivo may in part result from the increased induction of the apoptosis as well as inhibition of angiogenesis in the combined treatment. This suggestion is supported by the present findings. The more apparent apoptotic cells in the tumors treated with liposomal honokiol plus DDP was found in fluorescent in situ TUNEL assay, compared with the treatment with control groups, including PBS, liposome, honokiol or DDP alone. In addition, the combination of liposomal honokiol and DDP apparently reduced the number of vessels by immunolabeling of CD31 in the tissue sections, compared with control groups. DDP has been effective in inducing apoptosis in variety of tumor cell lines [13,16-18]. Its apoptosis-inducing effects are known to be correlated with DNA damage by forming DNA-Pt adducts and DNA strand breaks. It has been reported that DNA synthesis of human umbilical endothelial cells was in vitro inhibited by DDP in a dose-dependent fashion, and that rabbit corneal neovascularization in vivo was also suppressed by intravenous injection of DDP, suggesting that DDP might have an indirect anti-neoplastic effect through the suppression of neovascularization required for the tumor growth [19]. Recently, evidence indicated that honokiol directly triggered the apoptosis in variety of human cancer cells by the induction of caspase-dependent and -independent apoptosis [8,9]. It has been also reported that honokiol may be a potent inhibitor of angiogenesis and may inhibit angiogenesis by interfering with phosphorylation of VEGFR2 in human endothelial cells [4]. In addition, it was also reported that the invasion (MMP-9, IAM-1) and angiogenesis (VEGF) were down-regulated by honokiol [20]. Thus, we may speculate that besides the direct apoptotic effect of both liposomal honokiol and DDP on tumor cells themselves, the antiangiogenesis activity by liposomal honokiol and DDP may as well play a partial role in the antitumor activity by retarding or preventing adequate nourishment of tumors during their regrowth after a chemotherapeutic insult, resulting in tumor growth stasis. The other findings have shown that honokiol in the combination with low-dose docetaxel enhanced the inhibition of the growth and bone metastasis in prostate tumor [21] and enhanced the cytotoxicity induced by fludarabine, cladribine, or chlorambucil in B-cell chronic lymphocytic leukaemia (B-CLL) cells [22]. Furthermore, it was known that the combination of an antiangiogenic agent such as CXC Chemokine Ligand 10 [13], vascular endothelial growth factor receptor-2 antibody [23], squalamine [24], etc., with chemotherapy can enhance tumor growth inhibition. Taken together, these findings mentioned above also support our suggestion that the enhanced antitumor efficacy in the present study may in part result from the increased induction of the apoptosis as well as the increased inhibition of angiogenesis in the combined treatment.

It has been known that A549 cells have a mutant active Ras oncogene [25]. Our data suggest that honokiol alone had the antitumor activity against human lung cancer in A549 lung cancer xenograft model, and that the combination of honokiol with DDP can enhance the antitumor activity. It has also been reported that the mutant K-ras blocks the efficacy of targeted EGFR inhibitors, like iressa and tarceva [26]. Therefore, whether or not honokiol could sensitize A549 cells to these agents is an intriguing question to be further explored.

The preparation of the clinically available honokiol has been limited by its poor solubility. We have found that except for dimethylsulfoxide, honokiol was seldom solved in the other solvents available to the preparation of the drugs. However, there are concerns about using higher doses of dimethylsulfoxide, as it causes the dose-dependent hemolysis, it is harmful to the liver and kidneys, and it has an unpleasant odor for 48 hours after the administration [27-29]. In the present study, we encapsulated honokiol as PEG-PE modified liposome nanoparticle, since liposomes have been used previously as carriers for delivery of a variety of drugs, including antibiotic, antifungal, and cytotoxic agents [30,31]. In the present study, we found that honokiol-encapsulated liposome has shown the improvement in the solubility of honokiol and the prolongation of its circulation time in plasma. The management of unresectable lung cancers remains a major therapeutic challenge to medical oncologists [1,3]. The present study has demonstrated that liposomal honokiol had the inhibitory effect on the growth of human lung cancer in A549 lung cancer xenograft model, and that liposomal honokiol plus DDP induced the enhanced antitumor activity without adverse consequences found. Thus, our observations may have potential implications for the treatment of human lung cancer by liposomal honokiol plus DDP.

## Conclusion

In summary, our data suggest that that liposomal honokiol alone had the antitumor activity against human lung cancer in A549 lung cancer xenograft model, and that the combination of honokiol with DDP can enhance the antitumor activity, and that the enhanced antitumor efficacy in vivo may in part result from the increased induction of the apoptosis and the enhanced inhibition of angiogenesis in the combined treatment. The present findings may be of importance to the further exploration of the potential application of the liposomal honokiol alone or the combined approach in the treatment of lung carcinoma.

## Competing interests

The authors declare that they have no competing interests.

## Authors' contributions

QqJ, LyF carried out the animal experiment, participated in the sequence alignment and drafted the manuscript. GlY, WHG, and WlH carried out the immunohistochemical study. QqJ and LjC participated in the design of the study and performed the statistical analysis. YqW conceived of the study, and participated in its design and coordination. All authors read and approved the final manuscript.

## Pre-publication history

The pre-publication history for this paper can be accessed here:



## References

[B1] Khuri FR, Herbst RS, Fossella FV (2001). Emerging therapies in non-small-cell lung cancer. Ann Oncol.

[B2] Soria J-C, Jiang SJ, Khuri FR, Hassan K, Liu D, Hong WK, Mao L (2000). Overexpression of cyclin B1 in early-stage non-small cell lung cancer and its clinical implication. Cancer Res.

[B3] Vora SA, Daly BDT, Blaszkowsky L, McGrath JJ, Bankoff M, Supran S, Dipetrillo TA (2000). High dose radiation therapy and chemotherapy as induction treatment for stage III nonsmall cell lung carcinoma. Cancer.

[B4] Bai X, Cerimele F, Ushio-Fukai M, Waqas M, Campbell PM, Govindarajan B, Der CJ, Battle T, Frank DA, Ye K, Murad E, Dubiel W, Soff G, Arbiser JkL (2003). Honokiol, a small molecular weight natural product, inhibits angiogenesis in vitro and tumor growth in vivo. J Biol Chem.

[B5] Hirano T, Gotoh M, Oka K (1994). Natural flavonoids and lignans are potent cytostatic agents against human leukemic HL-60 cells. Life Sci.

[B6] Wang T, Chen F, Chen Z, Wu YF, Xu XL, Zheng S, Hu X (2004). Honokiol induces apoptosis through p53-independent pathway in human colorectal cell line RKO. World J Gastroenterol.

[B7] Hibasami H, Achiwa Y, Katsuzaki H, Imai K, Yoshioka K, Nakanishi K, Ishii Y, Hasegawa M, Komiya T (1998). Honokiol induces apoptosis in human lymphoid leukemia Molt 4B cells. Int J Mol Med.

[B8] Ishitsuka K, Hideshima T, Hamasaki M, Raje N, Kumar S, Hideshima H, Shiraishi N, Yasui H, Roccaro AM, Richardson P, Podar K, Gouill SL, Chauhan D, Tamura K, Arbiser J, Anderson KC (2005). Honokiol overcomes conventional drug resistance in human multiple myeloma by induction of caspase-dependent and independent apoptosis. Blood.

[B9] Yang SE, Hsieh MT, Tsai TH, Hsu SL (2002). Down-modulation of Bcl-XL, release of cytochrome c and sequential activation of caspases during honokiol-induced apoptosis in human squamous lung cancer CH27 cells. Biochem Pharmacol.

[B10] Konoshima T, Kozuka M, Tokuda H, Nishino H, Iwashima A, Haruna M, Ito K, Tanabe M (1991). Studies on inhibitors of skin tumor promotion, IX: neolignans from Magnolia officinalis. J Nat Prod.

[B11] The International Adjuvant Lung Cancer Trial Collaborative Group (2004). Cisplatin-based adjuvant chemotherapy in patients with completely resected non-small-cell lung cancer. N Engl J Med.

[B12] Folkman J (1995). Seminars in medicine of the Beth Israel Hospital, Boston. Clinical applications of research on angiogenesis. N Engl J Med.

[B13] Li G, Tian L, Hou JM, Ding ZY, He QM, Feng P, Jun WY, Xiao F, Yao B, Zhang R, Peng F, Jiang Y, Luo F, Zhao X, Zhang L, Zhou Q, Wei YQ (2005). Improved therapeutic effectiveness by combining recombinant CXC chemokine ligand 10 with Cisplatin in solid tumors. Clin Cancer Res.

[B14] Wang X, Wang Y, Geng Y, Li F, Zheng C (2004). Isolation and purification of honokiol and magnolol from cortex Magnoliae officinalis by high-speed counter-current chromatography. J Chromatogr A.

[B15] Yuan ZP, Chen LJ, Fan LY, Tang MH, Yang GL, Yang HS, Du XB, Wang GQ, Yao WX, Zhao QM, Bin Y, Rui W, Peng D, Wei Z, Wu HB, Xia Z, Wei YQ (2006). Liposomal quercetin efficiently suppresses growth of solid tumors in murine models. Clin Cancer Res.

[B16] Saris CP, Vaart PJ van de, Rietbroek RC, Blommaert FA (1996). In vitro formation of DNA adducts by cisplatin, lobaplatin and oxaliplatin in calf thymus DNA in solution and in cultured human cells. Carcinogenesis.

[B17] Faivre S, Chan D, Salinas R, Woynarowska B, Woynarowski JM (2003). DNA strand breaks and apoptosis induced by oxaliplatin in cancer cells. Biochem Pharmacol.

[B18] Zhong X, Li X, Wang G, Zhu Y, Hu G, Zhao J, Neace C, Ding H, Reed E, Li QQ (2004). Mechanisms underlying the synergistic effect of SU5416 and cisplatin on cytotoxicity in human ovarian tumor cells. Int J Oncol.

[B19] Yoshikawa A, Saura R, Matsubara T, Mizuno K A mechanism of cisplatin action: antineoplastic effect through inhibition of neovascularization. Kobe J Med Sci.

[B20] Ahn KS, Sethi G, Shishodia S, Sung B, Arbiser JL, Aggarwal BB (2006). Honokiol potentiates apoptosis, suppresses osteoclastogenesis, and inhibits invasion through modulation of nuclear factor-kappaB activation pathway. Mol Cancer Res.

[B21] Shigemura K, Arbiser JL, Sun SY, Zayzafoon M, Johnstone PA, Fujisawa M, Gotoh A, Weksler B, Zhau HE, Chung LW (2007). Honokiol, a natural plant product, inhibits the bone metastatic growth of human prostate cancer cells. Cancer.

[B22] Battle TE, Arbiser J, Frank DA (2005). The natural product honokiol induces caspase-dependent apoptosis in B-cell chronic lymphocytic leukemia (B-CLL) cells. Blood.

[B23] Klement G, Baruchel S, Rak J, Man S, Clark K, Hicklin DJ (2000). Continuous low-dose therapy with vinblastine and VEGF receptor-2 antibody induces sustained tumor regression without overt toxicity. J Clin Invest.

[B24] Williams JI, Weitman S, Gonzalez CM, Jundt CH, Marty J, Stringer SD, Holroyd KJ, Mclane MP, Qiming C, Zasloff M, Von HD (2001). Squalamine treatment of human tumors in nu/nu mice enhances platinum-based chemotherapies. Clin Cancer Res.

[B25] Aoyama Y, Avruch J, Zhang XF (2004). Nore1 inhibits tumor cell growth independent of Ras or the MST1/2 kinases. Oncogene.

[B26] Viloria-Petit AM, Kerbel RS (2004). Acquired resistance to EGFR inhibitors: mechanisms and prevention strategies. Int J Radiat Oncol Biol Phys.

[B27] Muther RS, Bennett WM (1980). Ejects of dimethyl sulfoxide on renal function in man. JAMA.

[B28] Marshall LF, Camp PE, Bowers SA (1984). Dimethyl sulfoxide for the treatment of intracranial hypertension: a preliminary trial. Neurosurgery.

[B29] Rijtema M, Mosig D, Drukker A, Guignard JP (1999). The effects of dimethyl sulfoxide on renal function of the newborn rabbit. Biol Neonate.

[B30] Allen TM, Cullis PR (2004). Drug delivery systems: entering the mainstream. Science.

[B31] Batist G, Ramakrishnan G, Rao CS, Chandrasekharan A, Gutheil J, Guthrie T, Shah P, Khojasteh A, Nair MK, Hoelzer K, Tkaczuk K, Park, Lee LW (2001). Reduced cardiotoxicity and preserved antitumor efficacy of liposome-encapsulated doxorubicin and cyclophosphamide compared with conventional doxorubicin and cyclophosphamide in a randomized, multicenter trial of metastatic breast cancer. J Clin Oncol.

